# The Influence of Tibial Positioning on the Diagnostic Accuracy of Combined Posterior Cruciate Ligament and Posterolateral Rotatory Instability of the Knee

**DOI:** 10.4055/cios.2009.1.2.68

**Published:** 2009-05-26

**Authors:** Young-Bok Jung, Chang-Hyun Nam, Ho-Joong Jung, Yong-Seuk Lee, Young-Bong Ko

**Affiliations:** Department of Orthopedic Surgery, Medial Center of Chung-Ang University, Knee Center, Seoul, Korea.; * Department of Orthopedic Surgery, Korea University, Ansan Hospital, Ansan, Korea.

**Keywords:** Posterolateral rotatory instability, Dial test, Thigh-foot angle

## Abstract

**Background:**

To determine if tibial positioning affects the external rotation of the tibia in a dial test for posterolateral rotatory instability combined with posterior cruciate ligament (PCL) injuries.

**Methods:**

Between April 2007 and October 2007, 16 patients with a PCL tear and posterolateral rotatory instability were diagnosed using a dial test. The thigh-foot angle was measured at both 30° and 90° of knee flexion with an external rotation stress applied to the tibia in 2 different positions (reduction and posterior subluxation). The measurements were performed twice by 2 orthopedic surgeons.

**Results:**

In posterior subluxation, the mean side-to-side difference in the thigh-foot angle was 11.56 ± 3.01° at 30° of knee flexion and 11.88 ± 4.03° at 90° of knee flexion. In the sequential dial test performed with the tibia reduced, the mean side-to-side difference was 15.94 ± 4.17° (*p* < 0.05) at 30° of knee flexion and 16.88 ± 4.42° (*p* = 0.001) at 90° of knee flexion. The mean tibial external rotation was 5.31 ± 2.86° and 6.87 ± 3.59° higher in the reduced position than in the posterior subluxation at both 30° and 90° of knee flexion.

**Conclusions:**

In the dial test, reducing the tibia with an anterior force increases the ability of an examiner to detect posterolateral rotary instability of the knee combined with PCL injuries.

Posterior cruciate ligament (PCL) injuries are on the rise due to the increasing incidence of sports related accidents, road traffic accidents and industrial accidents, and are usually accompanied by injuries to the other knee structures, particularly the posterolateral ones. The advancement of diagnostic methods, such as physical examinations and MRI, and the higher interest in PCL injuries have helped to raise detection rates.^1^-4) However, many cases still go undiagnosed. They commonly result from an external rotation injury during knee extension activities or a direct blow to the anteromedial knee, and generally occur in combination with an anterior cruciate ligament injury.

Proper treatment of acute posterolateral instability results in more successful outcomes than a surgical reconstruction for chronic posterolateral instability.^5^-7) A failure to detect the instability has significant impact on the clinical treatment results of a combined ligament injury. O'Brien et al.8) attributed the poor results of anterior cruciate ligament reconstructions to undetected or untreated posterolateral instability, which accounted for 15% of the total failures. Hence, the importance of a precise diagnosis of combined injury cannot be underestimated, considering its effect on the clinical outcomes. In the literature, the controversy with the dial test (tibial external rotation test) lies in whether the patient should be placed in the prone or supine position and whether an anterior or posterior tibial load should be applied.9) It is suspected that a dial test performed with the knee in an abnormal anatomical position might not reveal injuries to the posterolateral structures. Therefore, the aim of this study was to improve the efficacy of the test by evaluating the tibial external rotation according to the tibial positioning during the dial test, which is a common diagnostic tool for posterolateral rotatory instability and PCL injury.

## METHODS

Between April 2007 and October 2007, 16 patients (16 knees) with a PCL injury and posterolateral rotatory instability were enrolled in this study. The dial test was performed on patients in the supine position. The interval from the time of injury to diagnosis was less than 3 months in 3 knees (19%), between 3 and 6 months in 3 knees (19%), and more than 6 months in 10 knees (62%). The mean age was 30 years (range, 14 to 49 years). Twelve of the subjects were young and active patients less than40 years of age and 4 were older than 40 years of age. Most of the study population was male (14). The lesion was on the right side in 10 patients and on the left in 6. The injury mechanism included sports related accidents in 9 patients, traffic accidents in 5 (3 passengers and 2 pedestrians), and falls in 2. Nine combined injuries were found: 4 cases of lateral meniscus injury, 3 cases of a medial meniscus injury, 2 cases of a minor partial tear of the anterior cruciate ligament, and 4 cases of a cartilage defect. Medial collateral ligament injury was excluded from combined injuries because it can affect the external rotation of the tibia.

**Fig. 1:**
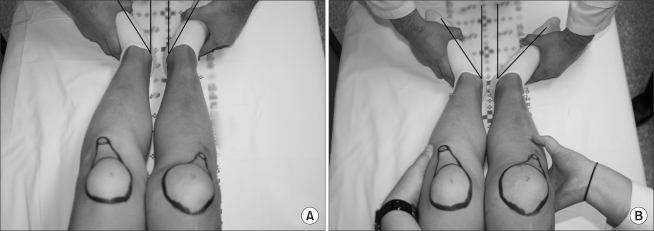
Thigh-foot angle was measured with an external rotation stress applied to the tibia at both 30° and 90° of knee flexion. Before applying the torque, a neutral force (A) and anterior force (B) was applied to the tibia.

For the diagnosis, the posterior and varus stress radiographs and MRI images were observed in the radiological examination and a posterior drawer test was performed to assess the PCL injury in the physical examination. Posterior displacement of the tibia was divided into 3 grades according to the difference between the affected and unaffected side: grade I indicated 3-5 mm of a side-to-side difference; grade II, 6-10 mm; and grade III, ≥ 11 mm. Six patients belonged to grade II and 10 patients to grade III. The posterolateral injuries were categorized according to the varus instability and rotatory instability. Varus instability was classified into 3 grades based on the difference in the lateral joint space opening between the injured leg and the other: grade I was defined as < 5 mm of a side-to-side difference; grade II as a 5-10 mm difference with a firm end point; and grade III as a more than 10 mm difference without a firm end point. No side-to-side difference was observed in 7 knees. There were 8 grade I knees and 1 grade II knee. Based on our encounters with cases where external rotation and varus instability were not in proportion, the external rotatory instability was assessed with respect to external rotation only: grade I was considered as less than a 5° increase in external rotation of the injured leg compared with the contralateral one; grade II was defined as a 5-10° side-to-side difference; and grade III was defined as a more than 10° difference.10) There were 10 grade II and 6 grade III knees. In the varus stress test, only one knee was considered to be higher than grade II. During the dial test, the patient was placed in the supine position and an assistant held the patient's knees to prevent external rotation. The measurements were performed with the knee at 30 and 90° of flexion both in posterior subluxation under a neutral force and in the reduced position under an anterior force. To evaluate the inter- and intraobserver error, each of the 2 examiners was asked to perform a couple of measurements. Due to the difficulty of measuring small angles, the minimum measurement unit was set to 5° (Fig. 1). Statistical analyses were performed using SPSS ver. 13.0. A paired-sample T-test and a Wilcoxon signed rank test were used to evaluate the angular changes for each patient. A *p* value < 0.05 was considered significant.

## RESULTS

**Fig. 2:**
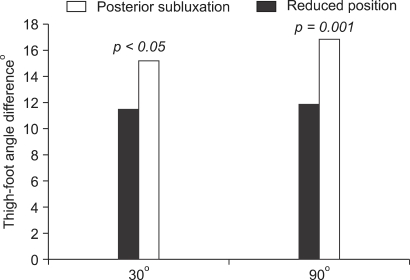
Difference in thigh-foot angle between 30° and 90° of knee flexion. The difference was greater in the reduced position than in posterior subluxation during test.

The dial test was performed on 16 patients and the measurements were considered reliable: the inter- and intraobserver reliability was low, 0.7865 to 0.8765. When the tibia was in posterior subluxation, the average (Table 1) thigh-foot angle at 30° of knee flexion was 35.00 ± 7.74° and 45.56 ± 7.89° on the uninjured and injured side, respectively. At 90° of knee flexion, the average (Table 1) thigh-foot angle was 35.93 ± 48.69° and 47.80 ± 8.75° on the uninjured and injured side, respectively. When the tibia was in the reduced position at 30° of knee flexion, the average (Table 1) thigh-foot angle was 35.93 ± 6.88° and 51.87 ± 8.34° on the uninjured and injured side, respectively, while at 90° of knee flexion, it was 37.18 ± 9.66° and 54.69 ± 8.05° on the uninjured and injured side, respectively. External rotation of the tibia was more noticeable when the tibia was reduced by an anterior force than maintained in posterior subluxation during the test (Fig. 2). At 30° of flexion of the knee, the side-to-side difference in the thigh-foot angle increased from 11.56 ± 3.01 to 15.94 ± 4.17° (*p* < 0.05), and the thigh-foot angle increased by 5.31 ± 2.86 on the lesion side. At 90° of flexion, it increased from 11.87 ± 4.03° to 16.87 ± 4.42° (*p* = 0.001) and 6.87 ± 3.59° more external rotation was observed on the affected side (Fig. 2). By the naked eye, the dial test appeared to indicate remarkable posterolateral rotation and subluxation when the tibia was anatomically reduced by an anterior force and the tibia-femur step off was restored than when it was posteriorly subluxated due to the gravity on the posterolateral aspect of the leg.

## DISCUSSION

In this study, the clinical test revealed significant rotatory instability of the knee in posterior subluxation and in the reduced position. The posterolateral drawer test and dial test for a diagnosis of posterolateral rotatory instability in PCL injuries with combined posterolateral structure injuries often produce misleading results if the tibia is posteriorly or posterolaterally subluxated during the test. This is because posterolateral subluxation of the tibia is difficult to palpate and the range of posterolateral rotation of the tibia is reduced. In contrast, when the test is performed with the proximal tibia in the reduced position, the increase in posterolateral rotation can be approximately 5° and ≥ 6° at 30° and 90° of flexion, respectively. Therefore, in order to obtain more precise results from the dial test, the proximal tibia should be pulled anteriorly to be in a normal anatomical position. According to Strauss et al.,11) when the posterior cruciate ligament, the popliteus tendon and the lateral collateral ligament were resected in this order, remarkable increases in the external rotation of the tibia were obtained from their cadaveric subjects at both 30° and 90° of flexion of the knee regardless of tibial positioning. In particular, they reported that 4.5-12° more external rotation of the tibia was achieved when the tibia was in the reduced position by an anterior force than a posterior force. In cadaveric studies, the reduction and posterior displacement of the tibia are created by a mechanical force. However, in the present study, as the study population consisted of live patients, an examiner was asked to hold the lateral and popliteal areas in order to elicit the posterior displacement and reduction in patients placed in the supine position.

Stabilization of the posterolateral aspect of the knee was obtained by the fibular collateral ligament and the popliteus complex, which is composed of the popliteus tendon and popliteofibular ligament. The fabellofibular ligament, which is also one of the posteolateral structures, is known to be of lesser value.^5^,^12^,13) Acute posterolateral instability can be diagnosed by a careful investigation of the development of tenderness in the posterolateral knee and the presence of a fibular head fracture or an arcuate fracture or a second fracture of the fibular head. Vascular damage should also be examined with care considering that a spontaneous reduction of tibial dislocation is common in cases of a severe combined ligament injury. In the diagnosis of chronic instability, the medical records, physical and radiological examination results, as well as the drive through signs should all be considered as there is no definite diagnostic method. Above all, posterolateral damage should be assumed to be present until demonstrated otherwise.14) In addition, anteromedial rotatory instability should be differentiated.1) Well-known physical tests include the external rotation recurvatum test, posterolateral drawer test, reserve pivot test, and dial test. With regard to the dial test, the authors observed the extent of rotation of the tibial tubercle during the test with the patient in the supine position because the ratio of tibial rotation to foot rotation is 1:3 and the test result can be affected by ankle and the foot deformities.10) In the radiological test, although stress radiography and MRI are helpful, the former makes it difficult to make precise comparisons due to rotation, and the latter can only be available when the magnetic field strength is > 1.5 T (tesla).1) Injuries to the posterolateral structures, albeit relatively uncommon, can lead to severe disorders. Because the lateral femoral condyle and lateral tibial plateau are convex structures, an injury to the posterolateral aspect might cause instability, albeit in the normal range, during walking and lateral compartment opening at foot strike.15) Various biomechanical functions of the posterolateral structures have been described in cadaveric studies involving desmotomy.^13^,^16^-20) These structures are vital for providing resistance to a varus force and an external rotation force: the lateral collateral ligament is against the former while the popliteus complex is against the latter.^15^,^16^,^21^,22) Grood et al.16) observed in their cadaveric study that external rotation of the tibia increased when the posterolateral structures were resected, which was more notable when the PCL was also removed.

Considering the association between the PCL and posterolateral structures, it is important to determine the impact of a PCL rupture on the posterolateral structure injuries in the physical test. In the case of grade III PCL injuries, the tibia was found to be subluxated more posteriorly than normal. Theoretically, when tibial external rotation is hampered by posterior subluxation, the opportunities for diagnosing posterolateral structure damage are reduced. On the other hand, more external rotation can be elicited if the tibia is reduced by an anterior force, or the patient is placed in a similar condition to the prone position, which increases the sensitivity of the dial test.

However, Pritsch et al.23) showed in their cadaveric study that removal of the medial collateral ligament produced similar results to that of the lateral collateral ligament during the dial test. Therefore, medial collateral ligament injuries can cause rotatory instability of the tibia by increasing the extent of external rotation.^20^,24) Therefore, in the physical examination of a PCL injury and combined posterolateral structure injuries, the valgus stress test should also be performed with the knee at 0 and 30° of flexion in order to determine the presence of a medial collateral ligament injury that can affect the clinical results. Among the many methods for classifying injuries to the posterolateral structures, no a single one was found to be reliable. Nevertheless, a more than 10° side-to-side difference and posterolateral subluxation of the tibial condyle are believed to be indications of surgery.^14^,^25^,26)

These features have been known to have limitations. Accordingly, clinical tests, including the varus stress test and the dial test, were carried out as there are no recognized objective tests that can lead to a diagnosis of posterolateral rotatory instability. In addition, research is currently being carried out on more precise measurement methods of tibial external rotation using CT with added coronal oblique views to obtain a more extended field of view of the posterolateral aspect with MRI. Therefore, in order to verify the objective efficacy of the index study, further studies will be needed to devise methods for a more precise diagnosis of posterolateral rotatory instability and analyze the extent of external rotation with the knee in posterior subluxation and a reduced position according to the level of posterior instability.

Injuries to the posterolateral structures of the knee often occur in combination with a PCL injury. Accordingly, precise diagnostic and proper treatment methods are required. This study can contribute to better reconstruction results by devising a more sensitive method for identifying combined injuries.

In this study the dial test was performed on patients with posterolateral cruciate ligament injury combined with posterolateral structure injuries. The results showed that tibial external rotation increased when the knee was in the reduced position, and posterior subluxation of the tibia was prevented when the patient was in the prone position. Therefore, the diagnostic efficiency of injuries to the posterolateral structures of the knee can be enhanced when the dial test is performed by placing the patient in the supine position and keeping the injured knee in the reduced position.

## Figures and Tables

**Table 1:**
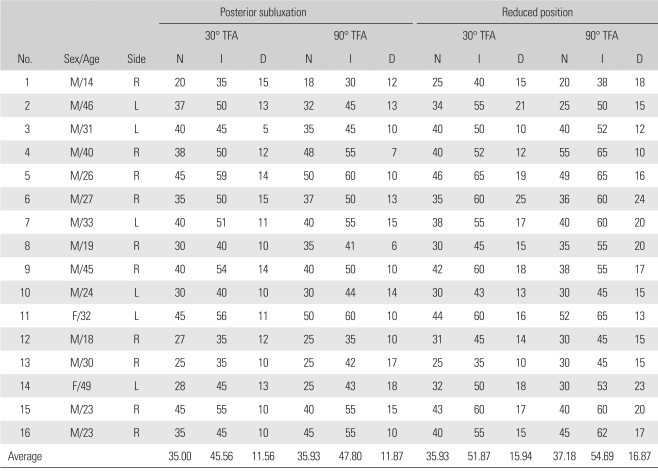
Patients' Data

TFA: Thigh-foot angle, N: Normal, I: Involved, D: Difference.
